# Challenges of assessing the burden of sepsis

**DOI:** 10.1007/s00063-023-01088-7

**Published:** 2023-11-17

**Authors:** Carolin Fleischmann-Struzek, Kristina Rudd

**Affiliations:** 1 Institute of Infectious Diseases and Infection Control, Jena University Hospital, Stoystr. 3, 07743 Jena, Germany; 2 Center for Sepsis Control and Care, Jena University Hospital, Jena, Germany; 3 The Clinical Research, Investigation, and Systems Modeling of Acute Illness (CRISMA) Center, Department of Critical Care Medicine, University of Pittsburgh, Pittsburgh, PA USA

**Keywords:** Mortality, Septic shock, Epidemiology, Incidence, Review, Mortalität, Septischer Schock, Epidemiologie, Inzidenz, Review

## Abstract

**Background:**

Sepsis is one of the most frequent causes of death worldwide, but the recording of population-based epidemiology is challenging, which is why reliable data on sepsis incidence and mortality are only available in a few, mostly highly-resourced countries.

**Objective:**

The aim of this narrative review is to provide an overview of sepsis epidemiology worldwide and in Germany based on current literature, to identify challenges in this research area, and to give an outlook on future developments.

**Materials and methods:**

Selective literature review. PubMed and Google Scholar were searched for current literature. The results were processed narratively.

**Results:**

Based on modeling studies or meta-analyses of prospective studies, global annual sepsis incidence was found to be 276–678/100,000 persons. Case fatality ranged from 22.5 to 26.7%. However, current data sources have several limitations, as administrative data of selected individual countries—mostly with high income—were used as their basis. In these administrative data, sepsis is captured with limited validity. Prospective studies using clinical data often have limited comparability or lack population reference.

**Conclusion:**

There is a lack of reliable data sources and definitions to monitor the epidemiology of sepsis and collect reliable global estimates. Increased policy efforts and new scientific approaches are needed to improve our understanding of sepsis epidemiology, identify vulnerable populations, and develop and target effective interventions.

## Abstract (de)

**Hintergrund:**

Sepsis stellte eine der häufigsten Todesursachen weltweit dar. Die Erfassung der bevölkerungsbezogenen Epidemiologie ist allerdings herausfordernd, weshalb es nur aus wenigen, meist hoch industrialisierten Ländern verlässliche Angaben zu Inzidenz und Sterblichkeit der Sepsis gibt.

**Zielsetzung:**

Ziel des narrativen Reviews ist es, auf Basis aktueller Literatur einen Überblick zur Sepsisepidemiologie weltweit und in Deutschland zu generieren, Herausforderungen in diesem Forschungsbereich zu benennen und einen Ausblick auf zukünftige Entwicklungen zu geben.

**Material und Methoden:**

Selektiver Literaturreview. Es wurde in PubMed und Google Scholar nach aktueller Literatur gesucht. Die Ergebnisse wurden narrativ aufbereitet.

**Ergebnisse:**

Basierend auf Modellierungsstudien oder Metaanalysen prospektiver Studien fand sich eine weltweite Sepsisinzidenz von 276–678/100.000 Menschen jährlich. Die Sterblichkeit lag zwischen 22,5 und 26,7 %. Beide Datenquellen haben aber relevante Limitationen, da insbesondere administrative Daten einzelner Länder – meist mit hohem Einkommen – als Basis verwendet wurden. In diesen administrativen Daten ist Sepsis mit eingeschränkter Validität abbildbar. Prospektive Studien unter Nutzung klinischer Daten sind häufig nur eingeschränkt vergleichbar und haben oft keinen Populationsbezug.

**Schlussfolgerung:**

Es fehlt an verlässlichen Datenquellen und Definitionen, um die Epidemiologie der Sepsis sicher zu überwachen und globale Schätzungen zu erheben. Zudem bedarf es vermehrter politischer Anstrengungen und neuer wissenschaftlicher Ansätze, um das Verständnis der Sepsisepidemiologie zu verbessern, vulnerable Populationen zu identifizieren sowie effektive Interventionen zu entwickeln und zielgerichtet zu implementieren.

Sepsis is the final common pathway of death from infection and a public health challenge for health care systems all over the world [1]. Although there are increasing numbers of studies examining the incidence of sepsis at the population level in individual countries, estimating the *global burden *of sepsis and sepsis incidence *on a population level* remains challenging. This is partly because comprehensive sources to generate such data are lacking, but also because existing population-level studies differ in their methodology and have methodological limitations, e.g., by their specific database or design, restricting the comparability and generalizability of their results. In addition, data from low- or middle-income counties (LMICs) are still scarce [2]. Improved understanding of sepsis epidemiology can help to identify vulnerable populations and target approaches for sepsis prevention. It also can support policy makers in resource allocation and inform future research focused on emerging pathogens, sepsis phenotypes, or specific patient populations. Therefore, the following review aims (i) to describe the current evidence on sepsis epidemiology on a population-level worldwide and in Germany, (ii) to analyze challenges in assessing this epidemiology, and (iii) to give an outlook on future research priorities. Underlying literature was obtained by selective literature search in PubMed and Google Scholar, including data published in the last 10 years, and summarized narratively.

## Assessing the global burden of sepsis

In the last decade, several major advances were made in our understanding of global sepsis epidemiology. Primary sources of this improved data were a new modeling approach using the Institute for Health Metrics and Evaluation’s (IHME) Global Burden of Disease (GBD) study, systematic synthesis of existing epidemiological studies among adults with sepsis, and a multinational point-prevalence study of maternal sepsis.

### Estimating the global burden of sepsis in the GBD framework

The 2020 IHME Global Burden of Sepsis study estimated 48.9 million (95% uncertainty interval [UI] 38.9–62.9 million) incident sepsis cases worldwide in 2017, which is equivalent to 677.5 (95% UI 535.7–876.1) cases per 100,000 age-standardized population [3]. Half of cases were found in children, the majority in those in the age of under five years. Sepsis was related to 19.8% of global deaths—11.0 million (95% UI 10.1–12.0 million) in 2017. According to the Global Burden of Sepsis study, both the age-standardized sepsis incidence and sepsis-associated morality rate decreased between 1990 and 2017 by 37.0% and 52.8%, respectively [3]. The study was the first attempt to leverage the GBD resources to generate estimates on sepsis epidemiology, as sepsis—always caused by an underlying infection—previously was not reported individually in the GBD report, rather sepsis deaths were counted under the patients’ underlying disease. Using multiple cause-of-death data from 109 million individual death records from four countries and 8.7 million hospital records from ten high- and middle-income countries, the 2020 IHME Sepsis study modeled the percentage of sepsis-related deaths by each underlying GBD cause of death for every country worldwide, adjusting for health-care access and quality. This methodology makes the results vulnerable to the imprecision of administrative coding in the primary data sources and may explain differences to primary studies examining the population-level burden of sepsis in individual countries (see below) [4]. However, the GBD offers important opportunities to improve our understanding of sepsis epidemiology by using a uniform methodologic approach across a time span of 27 years, all age groups, and 195 locations worldwide. Subsequent analyses of GBD data, for example, identified 33 bacteria as the underlying cause of global sepsis deaths (56.2%). Leading pathogens were *Staphylococcus aureus, Escherichia coli, Streptococcus pneumoniae, Klebsiella pneumoniae*, and *Pseudomonas aeruginosa* [5]. Of note, more than 6 million deaths were estimated to be a result of lower respiratory tract infections, bloodstream infections, or intra-abdominal infections, which underlines the preventive potential by vaccination and vaccine development, hygiene measures, and optimized access to and use of antibiotics [5].

### Evidence from population-level studies is restricted to middle- and high-income country data

Based on 51 studies from high- and middle-income countries, a recent systematic review and meta-analysis estimated a lower global incidence of 189 hospital-treated sepsis cases per 100,000 persons, with an in-hospital case fatality of 26.7% [1]. The estimated incidence of ICU-treated sepsis from 34 studies was 58 per 100,000 persons (in-hospital case fatality 41.9%). There was a considerably higher incidence of hospital-treated sepsis observed after 2008 (+46% compared to the overall time from 1979 onwards). As most studies included in the meta-analyses originated from high-income countries (46/51), their representativeness to the global population is limited. Furthermore, a substantial number of studies used administrative data to generate population-based estimates on sepsis incidence with inherent limitations (see below), and details on underlying pathogens, potential antimicrobial resistance, origin of infection, clinical treatments, and patient-relevant and long-term outcomes beyond hospital mortality remain unknown. The lower incidence rate in this study relative to the IHME Sepsis study may be explained by lower sepsis rates in high-income countries, inclusion of only hospitalized patients, restriction of data to studies of adults, and an underrepresentation of sepsis in administrative data [6]. Primary datasets in the meta-analysis mostly originated from high-income countries with aging populations, a high number of elderly patients with comorbidities, and an increasing use of invasive and complex treatments, which may lead to more health care-associated infections and sepsis [7]. In addition, improved sepsis awareness, capacity for diagnosis, and external incentives may have contributed to an increase in detection of cases and coding (= Will Rogers phenomenon [8]). The decrease in sepsis incidence from 1990–2017 found in the 2020 IHME Sepsis study may on the other hand be driven by a decreasing number of deaths from infectious diseases in LMICs [3]. Furthermore, improvements in sepsis prevention and treatment in countries with data input in the IHME Sepsis study influence the estimates obtained for all other countries and may project decreasing incidence and mortality rates in the absence of real improvements in other countries [4].

### Improved understanding of maternal sepsis prevalence

In 2020, the World Health Organization (WHO) Global Maternal Sepsis Study (GLOSS) Research Group published a major study of the 1‑week point prevalence and management of maternal infection in health facilities in 52 countries, many of which were LMICs [9]. This 1‑week prospective inception cohort study was conducted in 713 health facilities in 2017, and included 2850 pregnant or recently pregnant persons with suspected or confirmed infection. The study found that 70.4 (95% confidence interval [CI] 67.7–73.1) hospitalized persons per 1000 live births had a maternal infection, and 10.9 (9.8–12.0) per 1000 presented with severe infection-related maternal outcomes. Among persons with severe maternal outcomes, hospital mortality rate was 6.8%, and more than half of hospital deaths were associated with maternal infection. The highest prevalence and hospital case fatality rates were in LMICS. This study is the first to provide global data on the frequency and management of maternal infections, and unlike many previous studies included data for both direct (obstetric) and indirect (non-obstetric, e.g., pneumonia, malaria) maternal infections. While this study did not explicitly report maternal sepsis separately from uncomplicated acute maternal infections, data on severe maternal outcome provide some insight into the minimum estimated number of patients with maternal sepsis. This study suggests that the prevalence of maternal sepsis is higher than previously thought, consistent with the IHME Sepsis estimates that maternal sepsis is among the leading causes of sepsis incidence and sepsis-associated mortality globally [3].

## Burden of sepsis in Germany

**Fig. 1:**
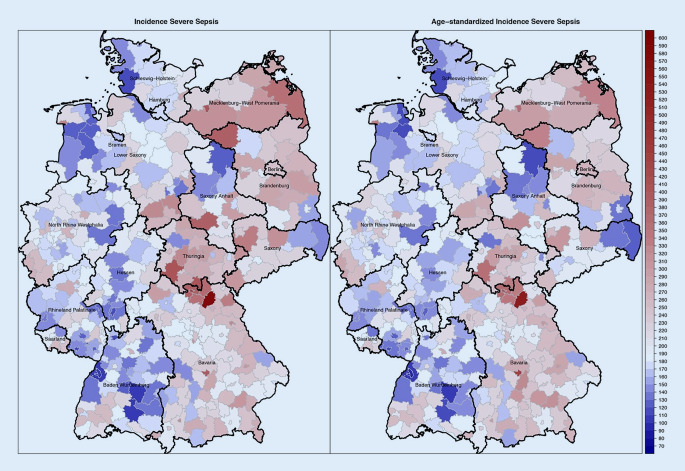
Distribution of crude and age-standardized explicitly defined sepsis incidence identified by ICD-10-GM codes R65.1 (severe sepsis) and R57.2 (septic shock) across German districts based on an analysis of the nationwide DRG statistics. From [13] (Geodata and shapefiles for creating maps of Germany in R were retrieved from https://gadm.org/)

Epidemiological data on the burden of sepsis in Germany was obtained from two major prospective point prevalence studies among intensive care unit (ICU) patients and nationwide analyses of hospital discharge data. The first point prevalence study included 454 ICUs from a representative nationwide sample of 310 hospitals in 2003, and estimated a sepsis prevalence of 11.0% among ICU patients [10]. This point prevalence was extrapolated to a population-based incidence of 76 cases of ICU-treated sepsis per 100,000 population in Germany. Ten years later, the INSEP study observed a sepsis point prevalence of 17.9% among 11,883 ICU patients from 133 ICUs and estimated an incidence of 11.64 sepsis cases per 1000 ICU days [11]. Hospital mortality ranged between 40.4% [11] and 55.2% [10]. In addition, studies using hospital discharge data were conducted after 2015 in Germany [12–14]. These studies identified sepsis by International Classification of Disease (ICD) codes or code combinations among all hospitalizations in acute care hospitals in Germany using the nationwide Diagnostic-Related Group (DRG) statistics of the Federal Statistical Office. They concluded an incidence nearly twice as high as the point prevalence study from 2003 (158 vs. 76/100,000), but comparable hospital mortality as the INSEP study (41.7% vs. 40.4%). Moreover, incidence rates were found to increase over time in hospital discharge data, and a considerable regional variation in sepsis incidence and mortality was observed between districts and federal states (Fig. 1), particularly when considering differences in age structure between districts [13]. Some of this variation can be explained by contextual determinants: residence in districts with lower socioeconomic status (e.g., less education) and greater distance to pharmacies as surrogates for the density of medical infrastructure was found to be associated with an increased sepsis incidence in Germany, but also differences in coding, sepsis awareness and local campaigns may contribute to the observed variation [13].

## Burden of sepsis is not only about acute illness

When discussing the burden of sepsis, it has to be acknowledged that for many sepsis survivors, sepsis is a life-changing event and comes along with a myriad of long-term sequelae [15]. In a healthcare claims-based study using complete records from 24 million insurance holders of the German health insurance AOK, three out of four sepsis survivors were affected by new mental, cognitive, or physical domain conditions in the first year after sepsis [16]. For example, 31.5% of survivors with no prior need for care had new care needs [16]. In particular, pre-existing comorbidities increased the risk for adverse outcomes after sepsis [17]. Long-term mortality in the first 12 months after discharge was 30.7% [16]. About 25% of survivors who were previously employed had not returned to work after one year. This adds to the burden of acute morbidity and mortality, although it is still under discussion to which degree long-term sequelae are sepsis-specific. Compared to matched survivors after other nonsepsis critical illness, patients with sepsis have higher healthcare resource use and costs but similar health-related quality of life [18]. Some long-term sequelae, such as cardiovascular disease [19] and all-cause mortality in the first 10 years after an episode of sepsis [20], occurred with higher frequency than in control populations.

## Sepsis epidemiology and COVID-19

In 2020, one out of three coronavirus disease 2019 (COVID-19) patients treated on general wards fulfilled the clinical criteria for sepsis according to a systematic review and meta-analysis [21]. The COVID-19 pandemic may have therefore considerably added to the burden of sepsis; and survivors of COVID-19 and sepsis endure similar long-term sequelae except for selected consequences such as thromboembolic manifestations which occur with higher frequency in COVID-19 survivors [22]. On the other hand, the incidence of non-COVID-19 sepsis may have been lower during the pandemic due to the reduction of other respiratory tract infections, and postsurgical sepsis [23]. Future iterations of the IHME Sepsis study and other sepsis epidemiology studies will provide more insight into trends in sepsis incidence and sepsis-associated mortality in the face of the COVID-19 pandemic.

## Challenges in assessing the burden of sepsis

### Limitations of existing research and databases

Despite the medical, social, and economic burden associated with sepsis, our understanding of the epidemiology of sepsis is still incomplete. This has several reasons: prospective cohort studies are often conducted with specific populations or small sample sizes, or use different designs and endpoints, hampering the comparability of findings and limiting the transferability to a population-level [2]. Diagnostic criteria for sepsis vary considerably between studies, as there is no single diagnostic test or marker for sepsis. The case definitions changed over the past three decades, most lately with the introduction of the Sepsis‑3 criteria [24], and clinical operationalization of the Sepsis‑3 definition continues to vary between studies. Furthermore, studies revealed high levels of discordance in the application of the sepsis criteria to clinical cases by clinicians, emphasizing the heterogeneity of the condition [25].

Large population-based studies often rely on administrative data such as hospital discharge data or cause of death statistics, in which sepsis patients are identified by specific ICD codes or code combinations. These data are not collected for research purposes and may be confounded by external incentives and changing coding policies or practices, making timely trends difficult to interpret [8]. Studies comparing the validity of sepsis coding with data from (electronic) patient charts concluded that the incidence of sepsis in still underestimated in hospital discharge data [26–28]. A recent US study found that sepsis incidence rates using clinical criteria in electronic health records (EHR) were relatively stable (+0.6% increase per year), whereas sepsis incidence per claims data increased by +10.3% per year [6]. Notably, the hospital admission rate (sepsis cases per 100 admissions) was 6.0% by EHR and 2.2% by administrative data (explicit sepsis codes), underlining the issue of incomplete capture in administrative data [6]. For Germany, the multicenter validation study OPTIMISE, which compared the accuracy of coding of sepsis in administrative data to reference standard diagnoses obtained by a chart review in 10 German hospitals, observed that the diagnosis of sepsis in administrative data had a high positive predictive value (76.9–85.7% depending on sepsis definition), but suffered from low sensitivity (26.8–38%) [29]. This led to an underestimation of hospital sepsis incidence (1.4% sepsis cases identified in administrative data vs. 3.3% identified in patient charts for severe sepsis‑1 among 100 admissions) and an overestimation of sepsis case fatality, as primarily cases with higher sepsis severity were coded correctly in administrative data [29]. Similar observations were made in a study from Sweden in terms of incidence (hospital admission rate: 4.1% sepsis‑3 in patient charts vs. 1.0% explicit coding in administrative data, incidence: 747 vs. 287 per 100,000 population) and mortality (20.1% vs. 23.2%) [28]. This underlines the need to consider these methodological limitations in the interpretation of estimates drawn from administrative data. Another major challenge is that administrative data are available mostly in high- and middle-income countries, which contributes to the research and knowledge gap between low- and high-middle-income countries.

### Challenges in sepsis epidemiology research in Germany

There are several specifics of the German health care system that make a comprehensive assessment of the burden of sepsis even more challenging. First, a considerable proportion of hospitals rely on paper charts for documentation, and the adoption of EHR in German hospitals is still incomplete. While countries such as the US or Norway increasingly use EHR data for epidemiological sepsis surveillance and benchmarking of sepsis outcomes between hospitals [30], Germany lacks the infrastructures for the implementation of EHR-based algorithms in many hospitals. Therefore, epidemiological studies and quality initiatives such as the German Quality Network Sepsis currently often base their analyses on administrative data such as hospital discharge data collected for reimbursement purposes in the German DRG system [31]. The future use of such data with ICD-based case identification, however, was challenged with the revision of ICD-10-German Modification (GM) sepsis codes in 2020, which omitted the use of the clinically defined codes R65.0 and R65.1 for sepsis, which was particularly a hurdle for coding sepsis cases without or with a viral focus. To address that gap, new sepsis ICD-10-GM codes were introduced in 2023 that allow to code for viral and fungal sepsis, and to differentiate the nosocomial vs. community-acquired origin of sepsis (Table 1).

**Table 1:** ICD-10 German Modification (GM) Codes for Sepsis introduced in 2023, allowing to code sepsis due to underlying viral, protozoal or fungal infection. Detailed information on sepsis coding in Germany can be found in the coding guidelines of the German Quality Network Sepsis [35]

*New coding for sepsis caused by viruses, protozoa and fungi*	
B00.70	Sepsis due to herpes viruses
B34.80	Sepsis due to viruses, not elsewhere classified
B38.70	Sepsis due to Coccidioides
B39.30	Sepsis due to Histoplasma capsulatum
B40.70	Sepsis due to Blastomyces
B41.70	Sepsis due to Paracoccidioides
B42.70	Sepsis due to Sporothrix
B44.70	Sepsis due to Aspergillus
B45.70	Sepsis due to Cryptococcus
B46.40	Sepsis due to Mucorales
B48.80	Sepsis due to fungi, not elsewhere classified
B58.90	Sepsis caused by toxoplasma
B60.80	Sepsis due to protozoa, not elsewhere classified
*New coding for nosocomial/non-nosocomial sepsis origin.* *These codes should be reported as additional codes*	
U69.80!	Non-nosocomial sepsis Sepsis occurring before the third calendar day of hospital admission
U69.81!	Nosocomial sepsis Sepsis occurring on or after the third calendar day of hospital admission
U69.82!	Sepsis with unclear time of onset with reference to hospital admission
U69.83!	Non-nosocomial septic shock Septic shock occurring before the third calendar day of hospital admission
U69.84!	Nosocomial septic shock Septic shock occurring on or after the third calendar day of hospital admission
U69.85!	Septic shock with unclear time of onset with reference to hospital admission

With respect to sepsis mortality, the lack of a multiple cause of death statistics in Germany impedes the assessment of sepsis mortality. In the system of the death statistics, sepsis is considered an immediate or intermediate, not the underlying cause of death. Thus sepsis-related deaths often ‘hide behind’ deaths coded due to pneumonia or other underlying causes. Therefore, to date, we lack information on out-of-hospital sepsis deaths, which comprise around one out of ten sepsis deaths according to a US study [32].

## Future directions of sepsis epidemiology research

**Fig. 2:**
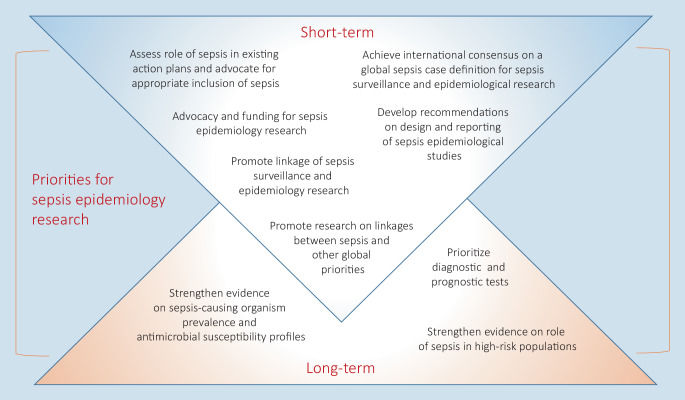
Global short- and long-term priorities for sepsis epidemiology research, modified based on the outcomes of the Technical Expert Meeting on Methodology for Sepsis Epidemiology Research [33]

To improve knowledge, reliable data and ways to operationalize sepsis definitions which are applicable in all resource settings are urgently needed. This requires sepsis to be included in national research and health care agendas, additional funding and strengthening of research capacities, and the support of global initiatives and programs, e.g., by linking sepsis epidemiology research with existing programs like on antimicrobial resistance or patient safety (Fig. 2; [33]). In Germany, this comes together with the call to foster the use of digital health care data. In the last few years, nationwide collaborations and platforms were initiated, which may also provide a path forward also for sepsis epidemiology research. First, the Medical Informatics Initiative (MII) funded by the German Federal Ministry of Education and Research aggregates and integrates electronic health care data across multiple entities and sites and make them available for research purposes [34]. On the other hand, nationwide data of all German health insurances will be made available in a research data center in the future; linkage with EHR is planned (https://www.forschungsdatenzentrum-gesundheit.de). This project of the German Federal Ministry of Health may foster the use of nationwide health claims data and their linkage with patient-level clinical data. Above that, also the quality assurance measures “Diagnostics, Therapy and Follow-up of Sepsis” of the Institute for Quality Assurance and Transparency in Healthcare will generate new insights and a benchmarking for sepsis care in Germany (https://iqtig.org/qs-verfahren/qs-sepsis/).

## Conclusions

Although sepsis is a common and life-changing disease, the burden is insufficiently understood. Intensified research in this area is urgently needed, as well as improved datasets to established reliable tools to measure and monitor sepsis incidence, case fatality and mortality. This can help inform health policy, healthcare provision and research efforts, and guide the implementation of novel treatments and interventions to improve the care of patients with sepsis and sepsis survivors.

## Declarations

For this article no studies with human participants or animals were performed by any of the authors. All studies mentioned were in accordance with the ethical standards indicated in each case.

The supplement containing this article is not sponsored by industry.
